# Histopathologic Validity and Clinical Reliability of Wolters Classification of Pulpitis: An Exploratory Study

**DOI:** 10.1111/iej.70213

**Published:** 2026-06-29

**Authors:** Çağla Fatmanur Yılmaz, Emre Barış, Ozlem Ilk, Güven Kayaoğlu

**Affiliations:** ^1^ Department of Endodontics, Faculty of Dentistry Gazi University Ankara Türkiye; ^2^ Department of Oral Pathology, Faculty of Dentistry Gazi University Ankara Türkiye; ^3^ Department of Statistics Middle East Technical University Ankara Türkiye

**Keywords:** concordance, dental pulp, diagnosis, endodontics, precision, validation study

## Abstract

**Aim:**

Wolters' classification of pulpitis has been proposed to classify pulpitis into 4 categories (initial, mild, moderate, severe), aiming to guide clinicians to implement the relevant vital endodontic treatment. The main objective of this study was to test the histologic validity and clinical reliability of the Wolters classification.

**Methodology:**

For the validity, before extraction, 52 teeth with pulpitis were examined and classified by one examiner into the appropriate Wolters category. After extraction and histological processing, a blinded pathologist examined the sections and assigned each tooth to a Wolters category. For the reliability, 140 patients with pulpitis were clinically and radiographically examined by two examiners, and pulpal and periapical diagnoses were made independently according to the Wolters and American Association of Endodontists (AAE) classifications. Finally, the diagnoses were unmasked, and the agreement was measured using Cohen's Kappa, weighted Kappa, or intraclass correlation coefficient (ICC). The correspondence between the categories of the Wolters and AAE classifications was examined using the chi‐square test and Cramer's *V* statistics. Statistical significance was set at *p* < 0.05.

**Results:**

The ICC and Kappa analyses indicated poor to fair agreement for the Wolters classification. However, in a re‐analysis performed by defining the mild pulpitis category to include a range from no to mild inflammation, the *κ* coefficients (minimum *κ* coefficient of 0.4004; *p* < 0.001) and the ICC value of 0.623 (95% CI: 0.319, 0.791) demonstrated fair to good agreement. Highest sensitivity [0.63 (95% CI: 0.39, 0.82)] and specificity [0.86 (95% CI: 0.69, 0.94)] were found in the severe and mild pulpitis categories, respectively. Regarding the clinical reliability, weighted‐Kappa tests (minimum *κ* coefficient of 0.8118, *p* < 0.001) and the ICC value of 0.945 (95% CI: 0.923, 0.961) suggested that the interobserver agreement for the Wolters classification was almost perfect to excellent. The Wolters' initial pulpitis category and the moderate and severe pulpitis categories corresponded to the AAE's reversible and irreversible pulpitis categories, respectively. However, the mild pulpitis category was interpreted differently by the examiners.

**Conclusions:**

The Wolters classification demonstrated high clinical reliability; however, its histopathological validity appeared limited. Further research is required to better establish its validity.

## 1 Introduction

Traditional root canal treatment (pulpectomy), which involves removing the entire pulp and filling the root canal with artificial materials, is a technically difficult and costly treatment that makes the tooth prone to fracture (van der Sluis et al. 2013; Wolters et al. 2017; Ahmed et al. 2023; Duncan et al. 2023). However, if preserved alive, the pulp, with its physiological capabilities (i.e., sensory, formative, defensive), is a far superior tissue to artificial fillings.

Vital pulp treatments (VPT) are procedures that maintain the viability of the pulp, including indirect and direct pulp capping, as well as partial and full pulpotomy. VPT is not a new concept, but with increased interest in minimally invasive, biologically active approaches in endodontics, and especially with the advent of hydraulic calcium silicate cements (HCSC), it has come to the fore again. Currently, the use of HCSCs is considered the gold standard in VPT, and with their favourable biological, physical and chemical properties, high clinical success rates are obtained with their use (Asgary et al. 2015, 2018, 2022; Qudeimat et al. 2017; Taha et al. 2017; Eghbal et al. 2020; Careddu and Duncan 2021; Santos et al. 2021; Edwards et al. 2021; Alnassar et al. 2022; Zhang et al. 2022; Ruiz‐González et al. 2022; Duncan 2022; Singla et al. 2023; Esteve‐Pardo et al. 2023; Aldeen et al. 2023; Jiménez‐Martín et al. 2024).

Success in VPT largely depends on correct case selection, which relies on accurate pulpal diagnosis through clinical and radiographic examinations. Previous studies have shown either no correlation or only a partial correlation between clinical diagnostic data and histological findings (Seltzer et al. 1963; Garfunkel et al. 1973; Dummer et al. 1980; Cisneros‐Cabello and Segura‐Egea 2005; Giuroiu et al. 2015; Raoof et al. 2022; Dastpak et al. 2023; Dhillon et al. 2023). In contrast, few studies have found a high degree of concordance between clinical and histological diagnoses of healthy and inflamed pulps (Ricucci et al. 2014; Naseri et al. 2017). Particularly in one study, it was reported that healthy vital tissue was often seen in the radicular pulps of teeth clinically diagnosed with irreversible pulpitis (Ricucci et al. 2014). Such findings have constituted a basis for a new term ‘partial irreversible pulpitis’ (European Society of Endodontology 2019). Upon these findings, Wolters et al. (2017) proposed a new four‐category ordinal classification of pulpitis, relating the severity of clinical symptoms to the extent of pulpal inflammation (i.e., initial, mild, moderate and severe pulpitis). A clinical study using this classification found it to be a better discriminator for the clinical success of VPT than the classical American Association of Endodontists (AAE) classification (Careddu and Duncan 2021). Recent studies testing this new classification system also found that many teeth that would normally require conventional root canal treatment could be managed using VPT techniques (Jiménez‐Martín et al. 2024; Karrar et al. 2025).

In healthcare research, many variables of interest or outcomes are abstract concepts known as theoretical constructs (e.g., quality of life, anxiety, depression). The measurement of such constructs requires the use of reliable and valid tests or instruments (Kimberlin and Winterstein 2008). Reliability indicates that something can be measured consistently (e.g., get similar results each time you test), while validity means that you are accurately measuring the thing you want to measure (Fitzner 2007). In other words, validity is the extent to which an instrument measures what it claims to measure (Kimberlin and Winterstein 2008). A recent study (Karrar et al. 2025) tested the reliability and clinical validity of the Wolters classification (i.e., how clinical and radiographic examination findings fit the Wolters categories). However, histopathologic assessment is the gold standard, and histopathologic validation still lacks.

The primary objectives of this study were to test the histopathologic validity and clinical reliability of the new Wolters pulpitis classification system. The secondary objective was to determine the correspondences between the categories of the new Wolters pulpitis classification system and the traditional AAE pulpal classification system.

## 2 Materials and Method

This prospective diagnostic study was approved by the Clinical Research Ethics Committee of Gazi University Faculty of Dentistry (decision no: GUDHKAEK.2023.15/2, date: Aug. 10, 2023). The formulation of the research question and data collection were planned prior to clinical examinations (the index test) and histological evaluations (the reference test). All patients participating in this study signed an informed consent form. These forms were kept in a sealed envelope locked in a file cabinet accessible only to the investigators of the study.

### 2.1 Inclusion and Exclusion Criteria

Patient selection was conducted between August 2023 and May 2024 using a convenience sampling approach, based on referrals to the Gazi University dental clinics. Symptomatic patients either with restorative/endodontic or extraction indications for carious teeth with inflamed pulps, aged ≥ 18 years old, and consenting to the study were included. Exclusion criteria included inability to communicate, ASA classification ≥ 4, presence of another painful tooth or periodontal problem that could confound the diagnosis, non‐odontogenic tooth pain and long‐term use of a medication that could alter pain sensation or the inflammatory process (e.g., corticosteroids, antidepressants). The flowchart of the study is shown in Figure 1.

**FIGURE 1:**
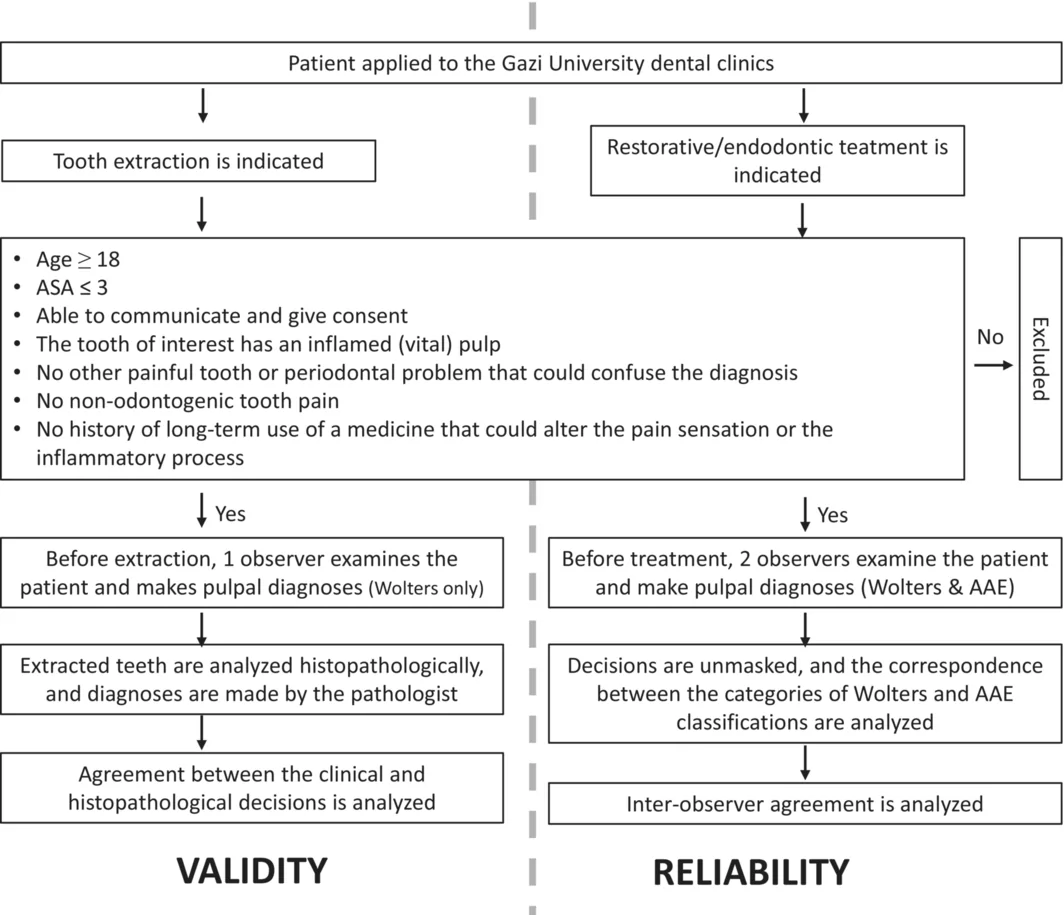
Flowchart of the study.

### 2.2 Sample Size Calculation

For the validity part, because the histopathological definition of the ‘initial’ category was not provided in the Wolters paper (Wolters et al. 2017), only the remaining three categories (mild, moderate and severe) were considered. The three categories assigned by the clinical and histopathological examiners comprised a total of 9 cells in the crosstab. With at least five volunteers required per cell, the number of volunteers was calculated to be 45 (extracted teeth with vital pulps). The ‘at least 5 observations per cell’ rule was applied as a conservative guideline to satisfy the assumptions of the chi‐square test. This criterion represents a rule of thumb rather than a strict statistical requirement. The classical Chi‐square Test of Independence relies on an asymptotic approximation to the chi‐square distribution, which becomes reasonably accurate when expected cell counts are ≥ 5 and, in larger contingency tables, when approximately 80% of cells have expected counts ≥ 5. Accordingly, sample size planning followed this guideline.

Additionally, a priori sample size calculation for Cohen's kappa used in validity analyses was performed using the method of Rotondi and Donner (2012), implemented in the kappaSize R package (Rotondi 2018). Under the assumptions of three possible outcome categories, two observers, a one‐sided significance level of 0.05, 80% power, testing *κ*_0_ = 0.5 (null hypothesis) versus *κ*_1_ = 0.8 (expected agreement), and nearly equal marginal outcome proportions, the required sample size was estimated to be 35. Therefore, the planned sample size of 45 was expected to provide power higher than 80%.

For the reliability part, the 4 Wolters categories (initial, mild, moderate, severe) assigned by 2 observers made up a total of 16 cells in the crosstab. At least 5 patients were required for each cell, and so the required number of patients was found to be 80, necessitating even distribution among the categories (approximately 20 patients for each category). However, a pilot sampling study showed a ratio of 3/6/8/3 for the incidence of the different categories. So, to reach at least 20 patients in each category, 140 patients were required.

A priori sample size calculation for Cohen's kappa used in reliability analyses with two possible outcomes for the AAE classifications and four outcomes for the Wolters classification was also performed. Under the assumptions of two observers, a one‐sided significance level of 0.05, 80% power, testing *κ*_0_ = 0.5 (null hypothesis) versus *κ*_1_ = 0.8 (expected agreement), and equal marginal outcome proportions, the minimum required sample sizes were estimated to be 52 for two outcomes and 29 for four outcomes. Therefore, the planned sample size of 140 was expected to provide power higher than 80%.

Additionally, five volunteers were required for the descriptive histologic analysis of the ‘initial pulpitis’ category.

In total, 190 patients (45 + 140 + 5) were required for this study.

### 2.3 Validity Assessment and the Histological Procedures

Teeth that had been decided to be extracted in the Oral and Maxillofacial Surgery clinic of the Faculty of Dentistry, Gazi University were clinically and radiographically examined by one of the researchers (C.F.Y), and a diagnosis was made according to the Wolters pulpitis classification before extraction (mild, moderate, or severe pulpitis) (Wolters et al. 2017). Reasons for the extraction included the tooth being a wisdom tooth, excessive crown damage and restorative difficulties, the patient's own request and prosthetic and orthodontic reasons. All the teeth had carious dentinal lesions of various depths. The reference standard for verifying the clinical pulpitis classifications was histopathologic examination, which is considered the gold standard. Immediately after extraction, the tooth was cut 2–3 mm from the root tip and stored in neutral buffered formaldehyde solution (10%). With clinical diagnoses concealed, each tooth was assigned a random 3‐digit code and sent to the Oral Pathology laboratory. Information on how many teeth there were in each class was also not provided to the laboratory. In the Oral Pathology laboratory, routine hard‐tissue decalcification and histological preparation procedures were followed (Khangura et al. 2021). Briefly, the teeth were decalcified in 10% (v/v) aqueous formic acid solution prepared from formic acid (98–100%, Cat. No: 100263.2500, Merck, Darmstadt, Germany), rinsed under running tap water for 4 h, dehydrated in ascending grades of ethanol (60%, 70%, 80%, 90%, 96% and 100%, respectively), cleared in xylene before embedding in melted paraffin, serially sectioned into 4 μm thick slices, and stained with haematoxylin and eosin (H & E) and Masson Trichrome. Then the sections were evaluated histopathologically by 1 blinded, experienced oral pathologist (E.B.: faculty member with 18 years of experience as a specialist) under a light microscope at ×40, ×100 and ×200 magnifications (Olympus BX51, Olympus Corp., Tokyo, Japan). The pathologist read and familiarized himself with the histological assumptions described by Wolters et al. (2017) before microscopic evaluation, and then assigned each tooth to an appropriate Wolters pulpitis category. After the histopathological evaluation was completed, the codes were removed, and the clinical and histopathological diagnoses were compared. In this part of the study, only teeth in the mild, moderate, and severe pulpitis categories were considered, as Wolters et al. (2017) provided histological definitions for only these categories.

Throughout the validity assessment, the clinical examiner and the oral pathologist were blinded to each other's decisions. However, with an exploratory approach, an extra 5 teeth clinically diagnosed as ‘initial pulpitis’ were extracted and evaluated descriptively. The clinical diagnoses of these teeth were not masked, nor were they included in the statistical analyses.

### 2.4 Reliability Assessment (Interobserver Agreement)

This part of the study was conducted at the general endodontic training clinic of the Faculty of Dentistry, Gazi University. Before commencing, the definitions of pulpitis categories according to AAE (American Association of Endodontists (AAE) 2020) and Wolters et al. (2017) classifications were reviewed, and various clinical cases were examined and discussed together by the examiners (C.F.Y: Observer 1, PhD student with 3 years of experience as a dentist, and G.K: Observer 2, faculty member with 16 years of endodontic experience as a specialist). With the commencement of the study, the patient complaints were listened to together by the examiners. Clinical and radiographic examinations were conducted independently by the two examiners, which included visual inspection, percussion and palpation tests and evaluation of digital periapical and panoramic radiographs. Since the thermal tests (ethyl chloride, ice) could be painful for some patients, they were applied only once and response was noted by both examiners. The examiners marked pulpal and periapical diagnosis categories for each patient on a chart and masked their decisions until the targeted number of patients was reached. Routine treatment procedures were then followed. In patients undergoing root canal treatment, if necrosis of the pulp (i.e., no bleeding from the pulp chamber) was detected during endodontic access, the patient was excluded from the study. The reason was that the Wolters classification intended to classify only teeth with pulpitis, and the symptom pattern of pulp necrosis could differ from that of pulpitis. In a few cases this occurred, and a new patient was recruited instead of the excluded patient. Finally, the decisions were unmasked, and agreements/disagreements were recorded.

### 2.5 Correspondence Between the Two Classification Systems

As a secondary aim of this study, the correspondence between the AAE pulpal diagnostic classification categories and the new Wolters classification categories was evaluated. In this part of the study, the observers were evaluated within themselves. Simply put, the match between the AAE and Wolters pulpitis diagnoses made by each observer was examined after unmasking the decisions. Since the examinations performed in the reliability analysis were sufficient for this purpose, a separate patient group was not examined.

### 2.6 Statistical Analyses

For the validity and reliability analyses, the agreement was measured using Cohen's Kappa, weighted Kappa, or intraclass correlation coefficient (ICC). Measures of diagnostic accuracy (specificity, sensitivity and predictive values) were calculated for the validity assessment.

The correspondence between the initial, mild, moderate and severe categories of the Wolters classification and the reversible and irreversible categories of the AAE classification was examined using the Chi‐square test and Cramer's *V* statistics. Cramer's *V* is a measure of the strength of association between two nominal variables. It ranges from 0 to 1 where 0 indicates no association and 1 indicates a strong association between the two variables. This measure takes the sample size and dimension of the table into account as well. Analyses were done using R Statistical Software (v4.5.0) (R Core Team 2025).

This article has been written according to the Preferred Reporting Items for Diagnostic Accuracy Studies in Endodontics (PRIDASE) 2024 guidelines.

## 3 Results

### 3.1 Descriptives

Each patient contributed with only one tooth to the study. For the validity part, histologic sections of 52 extracted teeth were analysed, of which six were considered as missing data and therefore excluded (carious lesion not found in the histologic sections: 2, inadequate histologic processing: 3, necrotic pulp: 1) (included *n* = 46; 38.7 ± 12.1 years; 35 female, 11 male). Additionally, histologic sections of another five extracted teeth were analysed for the definition of initial pulpitis (34.0 ± 6.2 years; 3 female, 2 male). And for the reliability analysis, 140 patients were included [average age (±SD): 38.8 ± 14.0 years; 99 female, 41 male].

### 3.2 Histopathologic Observations on the Teeth Diagnosed With Initial Pulpitis

In 3 of the 5 cases clinically diagnosed with initial pulpitis, the coronal and radicular pulps, composed of loose mixoid connective tissue, were totally healthy, with sound predentin and odontoblastic layers. However, in one case, limited local inflammation confined to the crown pulp, presenting with 10–20 lymphocytes neighbouring the odontoblast layer, was found. In another case, although the coronal pulp seemed healthy in general, a thick layer of tertiary dentine was present, confronting the deep dentinal carious lesion and exhibiting focal chronic inflammation beneath. The radicular pulp was healthy.

### 3.3 Validity

Not foreseen a priori, ‘no inflammation’ was found histologically in some of the teeth. Therefore, the data had to be presented in a 4 × 4 cross‐tabulation (instead of the previously planned 3 × 3) (Table 1; Condition 1). Although the Kappa analyses indicated fair agreement between clinical and histopathological diagnoses (Equal‐spaced weighted *κ* coefficient = 0.305, *p* < 0.001; Fleiss‐Cohen weighted *κ* coefficient = 0.365, *p* < 0.001), the ICC estimate (0.359) suggested poor agreement. Moreover, the confidence interval for the ICC (95% CI: −0.21 to 0.659) included zero, indicating that the agreement was not statistically significant.

**TABLE 1:** Cross‐tables matching clinical and histopathological diagnoses for two different conditions (*n* = 46), and the statistical results for each.

	Condition 1	Histopathological diagnosis (Wolters)			
		No inflammation^a^	Mild	Moderate	Severe
Clinical diagnosis (Wolters)	No inflammation^a^	0	0	0	0
	Mild	6	3	2	2
	Moderate	5	1	7	4
	Severe	2	1	3	10
	Equal‐spaced weighted *κ* coefficient = 0.305, *p* < 0.001; (fair agreement) Fleiss‐Cohen weighted *κ* coefficient = 0.365, *p* < 0.001 (fair agreement) ICC = 0.359 (95% CI: −0.21, 0.659) (poor agreement)				
	Condition 2	No to mild inflammation^a^		Moderate	Severe
	Mild	9		2	2
	Moderate	6		7	4
	Severe	3		3	10
	Equal‐spaced weighted *κ* coefficient = 0.4004, *p* < 0.001; (borderline between fair and moderate agreement) Fleiss‐Cohen weighted *κ* coefficient = 0.4467, *p* < 0.001 (moderate agreement) ICC = 0.623 (95% CI: 0.319, 0.791) (good agreement)				

^a^ Note that this term is not included in the original classification.

In this situation, we handled the data in an alternative way. Assumptively, we redefined the histopathological equivalent of the mild pulpitis category to include a range from ‘no inflammation’ to ‘mild inflammation’ (Condition 2). The rationale for this *post hoc* regrouping was:

1. A recent study has shown that grouping initial and mild pulpitis cases together in a 3‐cluster model improves the clinical validity of the Wolters classification (Karrar et al. 2025).
2. Histopathological observations we performed on teeth clinically diagnosed with initial pulpitis mostly showed no inflammation or only limited/superficial pulp inflammation. This suggests that the histological equivalence range for the initial + mild pulpitis cluster starts from absence of inflammation.

So, we reorganized the data and repeated the statistical analyses (Table 1; condition 2). In this case, the *κ* coefficients increased [(equal‐spaced weighted *κ* coefficient = 0.4004, *p*‐value < 0.001; (borderline between fair and moderate agreement); Fleiss‐Cohen weighted *κ* coefficient = 0.4467, *p* < 0.001 (moderate agreement))] The ICC value was 0.623 (95% CI: 0.319, 0.791), indicating good agreement between the clinical and histopathological diagnoses (Table 1; Condition 2). The sensitivity, specificity and the positive and negative predictive values are given in Table 2.

**TABLE 2:** Diagnostic metrics, sensitivity, specificity and positive (PPV) and negative predictive values (NPV) (95% CI) for Condition 2.

	TP	FP	TN	FN	Sensitivity	Specificity	PPV	NPV
Mild pulpitis	9	4	24	9	0.50 (0.29, 0.71)	0.86 (0.69, 0.94)	0.69 (0.42, 0.87)	0.73 (0.56, 0.85)
Moderate pulpitis	7	10	24	5	0.58 (0.32, 0.81)	0.71 (0.54, 0.83)	0.41 (0.22, 0.64)	0.83 (0.65, 0.92)
Severe pulpitis	10	6	24	6	0.63 (0.39, 0.82)	0.80 (0.63, 0.90)	0.63 (0.39, 0.82)	0.80 (0.63, 0.90)

Abbreviations: FN, false negative; FP, false positive; TN, true negative; TP, true positive.

Histologic sections representing the healthy and necrotic pulps are shown in Figure 2, whereas sections representing the categories of the Wolters classification are shown in Figure 3.

**FIGURE 2:**
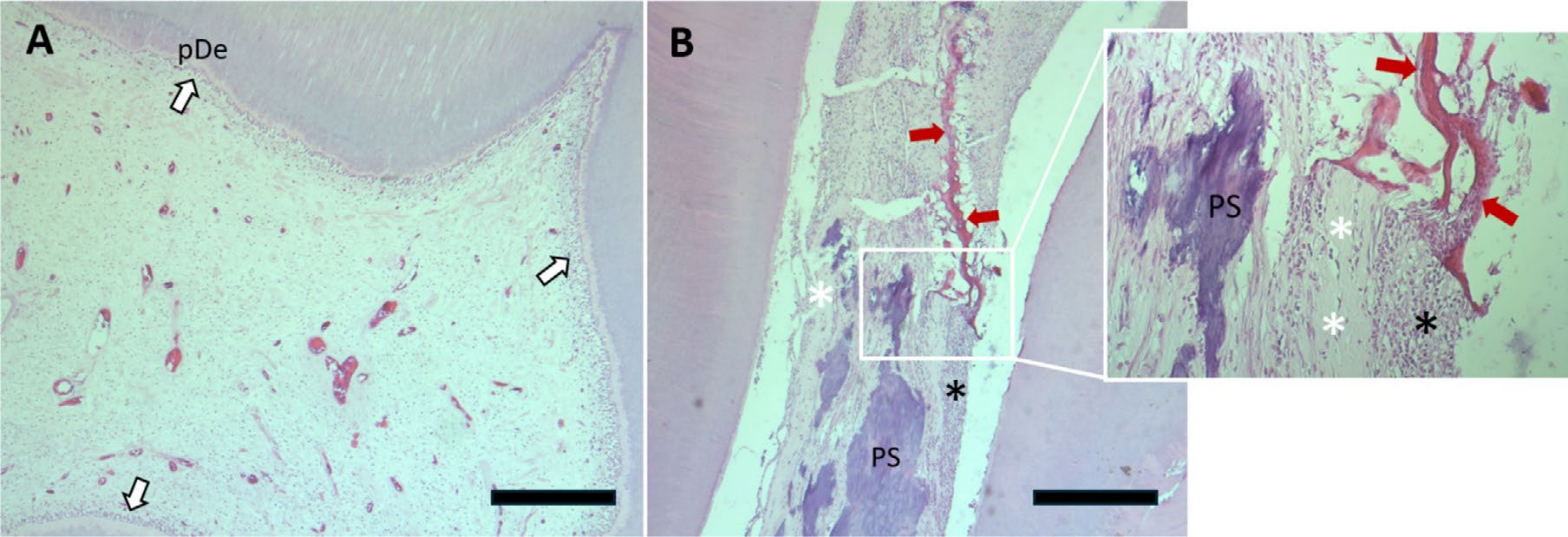
Histologic sections representative of healthy pulp and necrotic pulp. All photographs were taken using a camera mounted on the microscope (Leica DM4000 B, Leica Microsystems, Wetzlar, Germany). (A) Healthy pulp: The section includes the pulp horn at the right top corner and the floor of the pulp chamber at the left bottom corner. A thin layer of predentin (pDe) is visible along the inner border of the dentine. The adjacent odontoblastic layer is marked with white arrowheads. Blood vessels are seen within an uninflamed loose connective tissue. (H & E staining, scale bar = 500 μm; original magnification ×40). (B) Necrotic pulp: Partial necrosis extending into the radicular pulp is observed. Red arrowheads indicate necrotic fibrin foci. Black asterisks show areas with intense inflammatory cell accumulation, whereas white asterisks indicate apoptotic zones containing anucleate cells (PS: pulp stone, H & E staining, scale bar = 500 μm; original magnification ×40, and inset ×200).

**FIGURE 3:**
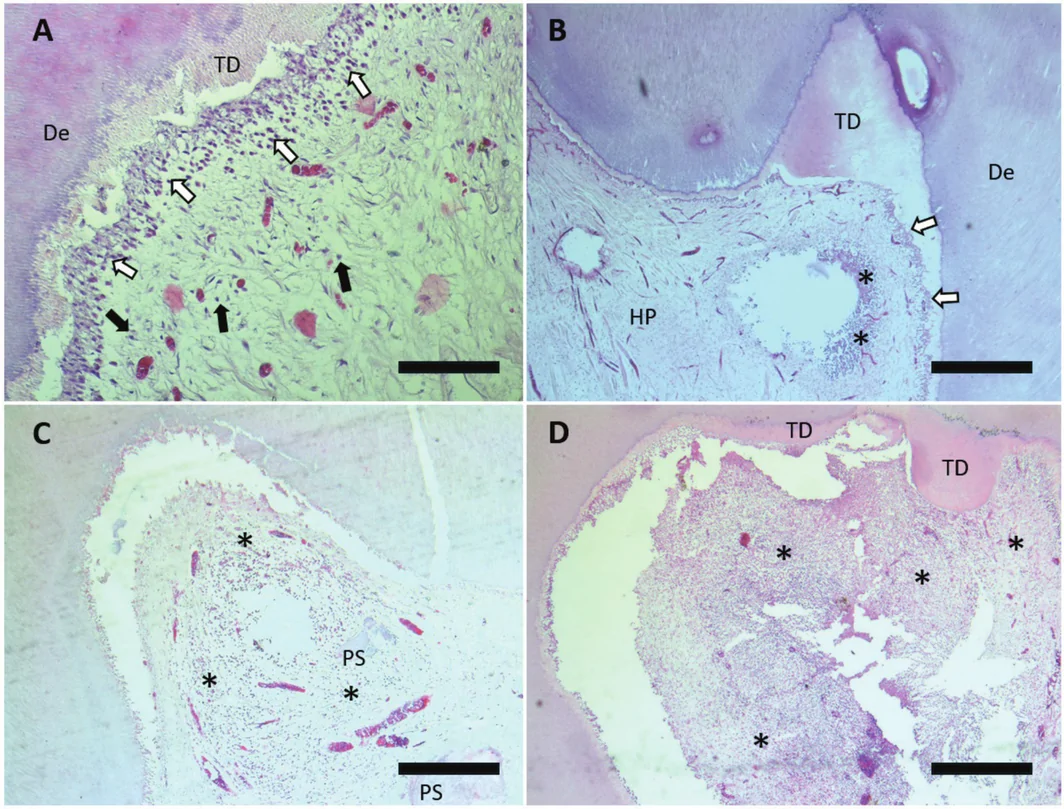
Histologic sections representative of each category of the Wolters pulpitis classification. (A) Initial pulpitis: Thickening of the odontoblastic layer (white arrowheads) and formation of tertiary dentine (TD) are observed with few inflammatory cells (lymphocytes; black arrowheads) (De: Dentine, H & E staining, scale bar = 100 μm; original magnification ×200). (B) Mild pulpitis: Localized inflammation with prominent tertiary dentine (TD) formation at the pulp horn. Black asterisks indicate the inflamed areas, and white arrowheads indicate the odontoblastic layer. Most of the pulp tissue appears healthy (HP: Healthy pulp; De: Dentine; H & E staining, scale bar = 500 μm; original magnification ×40). (C) Moderate pulpitis: Inflammation extending from the pulp horn over a wide area, with occasional formation of pulp stones (PS). Black asterisks indicate inflammatory cell accumulation. (H & E staining, scale bar = 500 μm; original magnification ×40). (D) Severe pulpitis. Section from coronal pulp showing intense, diffuse inflammation involving the entire pulp tissue (black asterisks). (TD, tertiary dentine; H & E staining, scale bar = 500 μm; original magnification ×40).

### 3.4 Reliability

According to the Wolters classification, overall, the two researchers agreed on 79.3% of the patients. This agreement ratio was 88.6% and 97.9% for the AAE pulpal and AAE periapical classifications, respectively. For the Wolters classification, all disagreements were observed in the one‐step disagreements (e.g., one researcher classified as moderate while the other classified it as severe). The pulpal and periapical classifications assigned by the observers are shown in Table 3 and Figure 4.

**TABLE 3:** Wolters and AAE pulpal and periapical classifications assigned by two observers are shown in a cross‐table. The diagonals of this table indicate the number of patients where two researchers agree, while the off‐diagonals represent the number of patients they disagree. Note that all disagreements are observed in the one‐step disagreements.

Wolters classification						
	Observer 1					
Observer 2		Initial	Mild	Moderate	Severe	Total
	Initial	22 (15.7%)	7 (5%)	0	0	29
	Mild	0	31 (22.1%)	8 (5.7%)	0	39
	Moderate	0	2 (1.4%)	38 (27.1%)	3 (2.1%)	43
	Severe	0	0	9 (6.4%)	20 (14.3%)	29
	Total	22	40	55	23	140

AAE pulpal classification				
	Observer 1			
Observer 2		Irreversible	Reversible	Total
	Irreversible	90 (64.3%)	16 (11.4%)	106
	Reversible	0	34 (24.3%)	34
	Total	90	50	140

AAE periapical classification				
	Observer 1			
Observer 2		Normal	SAP	Total
	Normal	37 (26.4%)	1 (0.7%)	38
	SAP	2 (1.4%)	100 (71.4%)	102
	Total	39	101	140

Abbreviation: SAP, symptomatic apical periodontitis.

**FIGURE 4:**
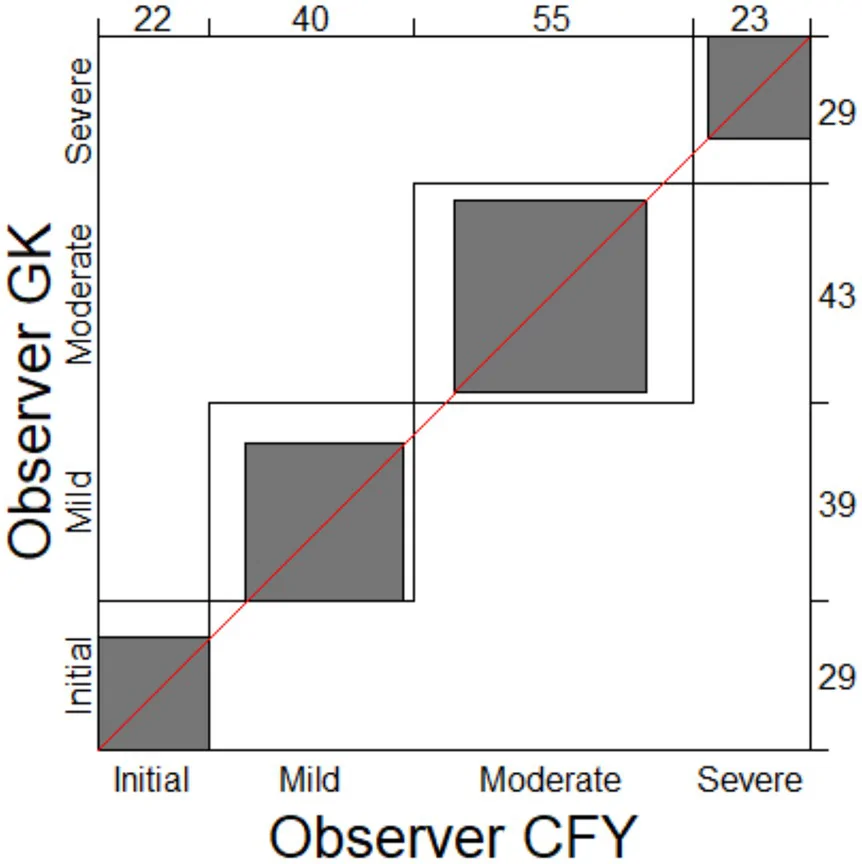
Agreement chart showing the concordance of the two observers for the Wolters classification. This graphic uses a quadratic weight, with the grey square denoting exact observed agreement and the white square, which includes a weighted contribution from off‐diagonal cells, denoting partial agreement.

For the Wolters classification, the unweighted‐Kappa test revealed substantial agreement (unweighted *κ* coefficient = 0.7115, *p* < 0.001), and the weighted‐Kappa test revealed almost perfect agreement between the two observers (equal‐spaced weighted *κ* coefficient = 0.8118, *p* < 0.001; Fleiss‐Cohen weighted *κ* coefficient = 0.8948, *p* < 0.001) (Landis and Koch 1977). The ICC value was 0.945 (95% CI: 0.923, 0.961), which was interpreted as excellent agreement between the two observers (Cicchetti 1994; Hallgren 2012).

For the AAE pulpal classification, the Kappa test revealed substantial agreement between the two observers (unweighted or weighted *κ* coefficients = 0.732, *p* < 0.001); and the ICC value was 0.861 (95% CI: 0.805, 0.9), which was interpreted as excellent agreement between the two observers.

For the AAE periapical classification, the Kappa test revealed almost perfect agreement between the two observers (unweighted or weighted *κ* coefficient = 0.946, *p* < 0.001).

### 3.5 Correspondence Between the Wolters and AAE Pulpitis Categories

Wolters and AAE pulpal classifications made by each observer were highly correlated; for Observer 1: chi‐square test value: 103.41, df = 3, *p* < 0.001 and Cramer's *V* = 0.86, (95% CI: 0.80, 0.92); and for Observer 2: chi‐square test value: 116.29, df = 3, *p* < 0.001 and Cramer's *V* = 0.91, (95% CI: 0.84, 0.98). The correspondence between the two pulpitis classification systems is shown in Table 4. According to this table, consistently, the Wolters' initial pulpitis category corresponded to the AAE's reversible pulpitis category, and the Wolters' moderate and severe pulpitis categories corresponded to the AAE's irreversible pulpitis category. But there was uncertainty for the mild pulpitis category. According to Observer 1, mild pulpitis was mostly reversible, while according to Observer 2, it was mostly irreversible pulpitis.

**TABLE 4:** Frequency table showing the correspondence between the Wolters and AAE pulpitis classification decisions for each observer.

	Irreversible	Reversible
Observer 1		
Initial	0	22 (100%)
Mild	12 (30%)	28 (70%)
Moderate	55 (100%)	0
Severe	23 (100%)	0
Observer 2		
Initial	0	29 (100%)
Mild	34 (87.2%)	5 (12.8%)
Moderate	43 (100%)	0
Severe	29 (100%)	0

### 3.6 *Post Hoc* Power Analysis

Approximate *post hoc* power values were calculated using the estimated weighted kappa coefficients and their asymptotic standard errors at 5% significance level and under the null hypothesis of no agreement. In addition, simulation‐based *post hoc* power estimates were obtained from 100 000 simulation runs under the same assumptions with approximate power calculations. Additionally, *post hoc* power calculations for chi‐square tests used in the correspondence analysis were performed according to the method described by Cohen (1988) using the pwr package in R (Champely 2020).

For the results of validity analyses, under Condition 1 in Table 1 (4 × 4 table), the approximate *post hoc* power estimates were 0.935 with equal‐spacing weights and 0.890 with Fleiss–Cohen weights. The corresponding simulation‐based power estimates were 0.927 and 0.863, respectively. For Condition 2 (3 × 3 table), the approximate *post hoc* power estimates were 0.949 using equal‐spacing weights and 0.931 using Fleiss–Cohen weights. The simulation‐based power estimates were 0.931 and 0.890 for equal‐spacing and Fleiss–Cohen weights, respectively. All of the *post hoc* power estimates for the results of reliability analyses given in Table 3 and for the results of correspondence analysis given in Table 4 were calculated as 1.

## 4 Discussion

The main findings of this exploratory study were that the original Wolters classification, as tested by two independent examiners, was reliable. Regarding validity, the original Wolters pulpitis categories were found to be poorly consistent with the described histological definitions (Wolters et al. 2017). But when a hypothetical regrouping was performed (the mild pulpitis category assumed to include no inflammation and initial inflammation as well), a fair to good level of agreement was found, indicating suboptimal histological validity.

The Wolters classification was developed to identify distinct levels of inflammatory pulpal disease and to guide appropriate vital conservative pulp treatment for each (Wolters et al. 2017). This will allow clinicians to gain several advantages by preserving the pulp alive, making the technically challenging pulpectomy procedure a last resort. In cases classified as irreversible pulpitis according to the traditional AAE classification, which necessitates complete pulp removal, Wolters' classification, with its theoretical foundations, still aims to preserve as much pulp tissue as possible. However, to be accepted as a scientific method, the Wolters classification must be both reliable and valid.

Interrater reliability (also called interobserver agreement) establishes the equivalence of ratings obtained with an instrument when used by different observers, and a reliable measurement requires consistency between the ratings of different raters (Kimberlin and Winterstein 2008). In the current study, the interobserver agreement for the Wolters classification ranged from substantial to excellent, as assessed using various statistical agreement analyses. The level of interobserver agreement in the current study was higher than that found in the Karrar study (2025), possibly because the current study had fewer observers (2 vs. 7 observers). On the other hand, the literature indicates that the interobserver reliability of the AAE pulpal/periapical classification has been scarcely studied. In a study where teeth were classified as diseased or disease‐free based on their pulpal and periapical states, a substantial interobserver agreement was found (unweighted *κ*: 0.74) (Mellin 2005). When compared using the unweighted *κ* coefficients, the interobserver agreement is similar between that in the Mellin study (2005) and in the Wolters and AAE pulpal classification parts of the current study.

In addition to unweighted Kappa tests (used for nominal variables), correlation coefficients and weighted Kappa tests were also applied in this study due to the ordinal structure of the classifications (de Raadt et al. 2021). Unlike unweighted tests, weighted tests take into account the degree of disagreement. Because there were only two variables in each AAE pulpal and periapical classification, the unweighted and weighted tests yielded the same result; however, for the 4‐level Wolters classification, the results differed. Two different weighted Kappa analyses were done. Between them, the equal‐spacing weights were defined by 1 − ∣*i* − *j*∣/(*r* − 1), where *i* and *j* denote the ratings/classification and *r* denotes the number of categories (*r* = 4 here), and the Fleiss‐Cohen weights by 1 − ∣*i* − *j*∣^2^/(*r* − 1)^2^. When there is perfect agreement between ratings or classifications, *i* = *j*, and both weighting schemes assign a value of 1. However, in other cases, the latter one attaches greater importance to near disagreements. Therefore, agreement values were found to be greater with the use of Fleiss‐Cohen weighted Kappa statistics.

Among different types of validity (e.g., construct, content, criterion), construct validity was tested in this study. Hypothetical constructs cannot be measured directly (such as the severity of pulpitis in this study). They can only be inferred from observations or phenomena that are considered indicators of the presence of the construct (Kimberlin and Winterstein 2008). Evaluation of construct validity requires examining the relationship of the measure being evaluated with variables known to be related or theoretically related to the construct measured by the instrument (Kimberlin and Winterstein 2008). Therefore, the instrument tested in this study (the Wolters classification) must be consistent regarding the clinical definitions of pulpitis severity and their corresponding pulp histopathology definitions. However, in this study, different statistical approaches showed fair to good agreement between the two. In endodontic studies, it is common to observe inconsistent results when comparing clinical findings and histopathological examinations (Seltzer et al. 1963; Garfunkel et al. 1973; Dummer et al. 1980; Cisneros‐Cabello and Segura‐Egea 2005; Giuroiu et al. 2015; Dastpak et al. 2023). Except for two studies reporting relatively higher accuracy (Ricucci et al. 2014; Naseri et al. 2017), this pattern persisted even when the AAE pulp classification terminology was tested (Raoof et al. 2022; Dhillon et al. 2023). The current study also does not provide sufficient evidence to support the histopathologic validity of the Wolters classification. However, the Karrar et al. (2025) study showed that the Wolters classification demonstrated good clinical validity overall, although the results of face and construct validity were suboptimal. In their study, a 3‐cluster model yielded the best diagnostic accuracy, which led the authors to reduce the categories to three, instead of four, by combining the initial and mild pulpitis groups into a single category and keeping the moderate and severe categories separate.

Unexpectedly, in this study, no inflammation was found in some of the samples classified as mild, moderate and severe pulpitis. A similar finding was reported in previous studies in which many of the extracted teeth diagnosed clinically with pulpitis exhibited no inflammation in their histologic analysis (Raoof et al. 2022; Dastpak et al. 2023). Speculatively, some neural changes resulting from caries progression may have caused the tooth to become painful before obvious inflammation developed. These changes may include neural sprouting, which leads to increased pulp innervation density, altered expression of voltage‐gated ion channels and transient receptor potential channels, and increased neuropeptide secretion by both neural and non‐neural cells of the dental pulp (Zhan et al. 2021). Patients' subjective responses to questions asked and tests administered during examination can also be the reason for a lack of precision (Hyman and Cohen 1984). Pain catastrophizing may be another explanation for the unexpected finding in this study. It refers to the tendency to describe a pain experience in more exaggerated terms than the average person, involving ruminating about the pain, magnifying it, and feeling helpless to manage it (Van Damme et al. 2002). Another explanation may be that the serial sections taken during histopathologic preparation missed the actually inflamed areas.

Furthermore, this study examined the correspondence between the categories of the two pulpal classification systems to determine how the traditional AAE pulpitis terminology translates to the Wolters pulpitis categories. Unanimously, Wolters' initial pulpitis category corresponded to the AAE's reversible pulpitis category, and Wolters' moderate and severe pulpitis categories corresponded to the AAE's irreversible pulpitis category. Yet, such a consensus was not achieved for the mild category, as one of the observers tended to classify mild pulpitis as reversible, while the other classified it as irreversible pulpitis. This disagreement may be because of a lack of sufficient diagnostic definitions (symptoms) provided in the original Wolters editorial (Wolters et al. 2017). By performing face validity analysis (subjective judgement of whether the instrument is a good measure or not), a possible lack of adequate preoperative symptoms/criteria was pointed out by Karrar et al. (2025), and modifications were proposed to improve the original Wolters classification. Similarly, another study that tested artificial intelligence for diagnosing pulpitis in adult patients using a modified Wolters classification suggested the need for additional, quantifiable clinical parameters to increase correct classification rates (Brizuela et al. 2026).

The strengths of this study include the histopathologic analysis (the accepted gold standard) carried out for assessing the validity of the new pulpitis classification. Additionally, the histopathologic definition of the initial pulpitis category, which was not provided in the original Wolters article (Wolters et al. 2017), was introduced in this study. Interestingly, another article also suggests “healthy pulp or local inflammation confined to the coronal pulp” to be the ‘likely histology’ of the initial pulpitis (Edwards et al. 2021). Our observational findings support their assumption.

On the other hand, one concern in this study relates to the *post hoc* regrouping of histopathologic categories and then repeating the validity testing. This approach was not pre‐specified, was introduced only after inspection of the data, and hence, can be regarded as exploratory. This was due to the unexpected absence of signs of inflammation in some of the histological sections, which prevented the targeted validity analysis from being performed as intended. To compensate for this, we redefined the histological definition of the mild pulpitis category. Based on our histological findings for initial pulpitis and the findings of a previous study (Karrar et al. 2025), which proposed combining the initial and mild pulpitis categories, we expanded the histological equivalence of the mild pulpitis category (referred to as Condition 2). Accordingly, we assumed that the absence of inflammation would be included within the mild pulpitis category. Looking at the improved, yet still wide, confidence interval values (compared to those that belong to the Condition 1), this *post hoc* approach appears to yield a more meaningful statistical result. The increased validity, resulting from reducing the number of Wolters categories to three (combining the initial and mild pulpitis categories), supports the findings of the Karrar study (2025), which showed that a 3‐cluster solution had the best clinical discriminative validity.

A limitation of the study was that the intra‐observer agreement, which includes the reproducibility of diagnoses made by the same observer at two different time points, could not be evaluated in this study. Because (i) it was unethical to re‐schedule a patient in a painful condition without commencing treatment, (ii) diagnosis could change in a relatively short time in pulpitis, (iii) observers would easily recall the first diagnosis unless a relatively long re‐evaluation appointment could be set. A potential source of bias in this study was selection bias, as the patients were not selected in a randomized manner. Particularly for the validity assessment, the tooth that best represented the appropriate inflammatory category was selected.

The generalizability of the findings is limited because the study was conducted in a single centre, using data from patients with a particular sociocultural background. Future research should include patients from diverse genetic and sociocultural backgrounds. And larger sample sizes should be considered to mitigate the effects of sample loss encountered in this study. Additionally, integrating specific biomarkers associated with inflammation severity into such diagnostic classification systems may enable clinicians to make a more objective and precise diagnosis of the pulp status (Brizuela et al. 2025).

In conclusion, the initial pulpitis category of the Wolters classification corresponded to the reversible pulpitis category of the AAE classification, and the moderate and severe pulpitis categories corresponded to the irreversible pulpitis category. However, the mild pulpitis category showed uncertainty as it could be interpreted variably as reversible or irreversible pulpitis by different observers. The reliability of the Wolters classification was found to be high and suitable for clinical use. The original assumptions of the Wolters pulpitis classification system showed a weak correlation with histology. However, when the definition of the mild pulpitis category was expanded to include a range from no to mild inflammation, coefficients of ‘clinical and histologic agreement’ increased (fair‐good). But, even in this case, the validity was suboptimal. Further research would be beneficial to confirm the histopathological validity of the Wolters classification.

## Author Contributions

All the authors have made substantial contributions to the manuscript. All the authors have read and approved the final version of the manuscript. **Çağla Fatmanur Yılmaz:** conceptualization, investigation, methodology, writing – original draft. **Emre Barış:** investigation, visualization, writing – review and editing. **Ozlem Ilk:** formal analysis, validation, visualization, writing – review and editing. **Güven Kayaoğlu:** conceptualization, investigation, methodology, writing – review and editing.

## Funding

The authors have nothing to report.

## Disclosure

Reporting guideline: This article has been written according to the Preferred Reporting Items for Diagnostic Accuracy Studies in Endodontics (PRIDASE) 2024 guidelines.

## Ethics Statement

This study was approved by the Clinical Research Ethics Committee of Gazi University Faculty of Dentistry (decision no: GÜDHKAEK.2023.15/2, date: August 10, 2023).

## Consent

All patients provided written informed consent before examination.

## Conflicts of Interest

The authors declare no conflicts of interest.

## Data Availability

The data that support the findings of this study are available on request from the corresponding author. The data are not publicly available due to privacy or ethical restrictions.
